# 1676. PK, Safety and Tolerability of VABOMERE^®^ (meropenem-vaborbactam) in Infants, Children and Adolescents

**DOI:** 10.1093/ofid/ofad500.1509

**Published:** 2023-11-27

**Authors:** Paula M Bokesch, Antonio C Arrieta, John S Bradley, Randall K Hoover, Sandra McCurdy

**Affiliations:** Melinta Therapeutics, LLC, Harvard, Massachusetts; Children's Hospital of Orange County, Orange, California, USA, Orange, California; University of California, San Diego, San Diego, CA; Melinta Therapeutics, Dix Hills, New York; Melinta Therapeutics, Dix Hills, New York

## Abstract

**Background:**

The worldwide spread of serine carbapenemases threatens carbapenems. VABOMERE (combination of meropenem and vaborbactam (MV), is a new class of beta-lactamase inhibitors including inhibition of *Klebsiella pneumoniae*. The PK, safety, and efficacy data of MV in adults support study in pediatrics.

**Methods:**

Phase 1, open-label, dose-finding study. Pediatric subjects, 3 mos. to < 18 yrs already on antibiotics received a single-dose weight based infusion of MV over 3 hr. Blood samples were drawn pre-dose, 3,4, and 6 hr. for PK parameters. Population PK model included weight as a covariate. Safety assessments included AEs, vital signs, safety labs, physical exams, infusion tolerance, ECG. A DSMB reviewed safety and PK data before enrolling next cohort.

**Results:**

MV was well-tolerated and safe in all age groups. Clearance per kg body weight of both M and V increased with decreasing body weight over all age groups suggesting an allometric relationship.

**Conclusion:**

Targeted AUC_0-8_ efficacious in adult trials ranges of 91.7-513 mg•hr/L (meropenem) and 131-494 mg•hr/L (vaborbactam) were achieved in all pediatric cohorts.

**Disclosures:**

**Paula M. Bokesch, MD**, Melinta Therapeutics: Advisor/Consultant **Antonio C. Arrieta, MD, FIDSA, FPIDS**, Astellas Pharma Global Development, Inc.: Advisor/Consultant|Astellas Pharma Global Development, Inc.: Grant/Research Support|Astellas Pharma Global Development, Inc.: Honoraria|Cumberland Pharmaceutical: Grant/Research Support|IDbyDNA: Advisor/Consultant|IDbyDNA: Grant/Research Support|Melinta: Grant/Research Support|Merck: Advisor/Consultant|Merck: Grant/Research Support|Nabriva: Grant/Research Support|Paratek Pharmaceuticals: Grant/Research Support|Pfizer, Inc: Advisor/Consultant|Pfizer, Inc: Grant/Research Support|Roche/Genentech: Grant/Research Support|The Medicine Company: Grant/Research Support **Randall K. Hoover, Ph.D.**, Melinta Therapeutics: Advisor/Consultant **Sandra McCurdy, MS**, Melinta Therapeutics: Employee

**Table 1.** Meropenem PK

	12-17 yrs 40 mg/kg n=8	6-11 yrs 40 mg/kg n=8	6-10 yrs 60 mg/kg n=4	2-5 yrs 60 mg/kg n=8	5-22 mos 60 mg/kg n=3	2-10 yrs 60 mg/kg n=7
Cmax ug/ml	62.4 ± 14.7	41.3 ± 17.4	41.1 ± 9.9	73.0 ± 34.8	62.6 ± 9.2	54.1 ± 22.6
Cmin ug/ml	1.4 ± 1.2	0.25 ± 0.25	0.11 ± 0.08	0.95 ± 1.19	0.68 ± 0.18	0.24 ± 0.28
AUC 0-8	209 ± 47.7	134 ± 58.7	127 ± 30.8	238 ± 115	199 ± 30.2	173 ± 70.0
t1/2 h	0.87 ± 0.17	0.61 ± 0.11	0.56 ± 0.07	0.71 ± 0.17	0.77 ± 0.03	0.60 ± 0.09
Vss L/kg	0.223 ± 0.062	0.291 ± 0.094	0.391 ± 0.099	0.303 ± 0.145	0.342 ± 0.052	0.457 ± 0.238
CL L.hr/kg	0.182 ± 0.059	0.344 ± 0.131	0.487 ± 0.155	0.313 ± 0.166	0.308 ± 0.044	0.533 ± 0.267

**Table 2.** Vaborbactam PK

	12-17 yrs. 40 mg/kg n=8	6-11yrs. 40 mg/kg n=8	6-10 yrs. 60mg/kg n=4	2-5 yrs. 60 mg/kg n=8	5-22 mos 60 mg/kg n=3	2-‹ 10 yrs 80 mg/kg n=7
Cmax ug/ml	60.8 ± 17.8	37.1 ± 17.3	45.6 ± 9.50	88.1 ± 45.2	105 ± 45.7	56.8 ± 24.4
Cmin ug/mL	2.8 ± 2.2	0.68 ± 0.72	0.27 ± 0.21	0.28 ± 0.12	2.86 ± 2.51	0.47 ± 0.46
AUC 0-8	209 ± 59.9	125 ± 61.5	144 ± 31.0	294 ± 154	164 ± 46.9	183 ± 76.1
T1/2 (h)	108 ± 0.23	0.78 ± 0.16	0.65 ± 0.083	0.82 ± 0.22	0.93 ± 0.11	0.69 ± 0.12
Vss L/kg	0.273 ± 0.085	0.412 ± 0.177	0.393 ± 0.070	0.280 ± 0.124	0.260 ± 0.103	0.479 ± 0.21
CL L/hr/kg	0.180 ± 0.06	0.384 ± 0.16	0.425 ± 0.12	0.251 ± 0.12	0.199 ± 0.09	0.483 ± 0.19

